# Intraneobladder Hem-o-Lok Migration with Stone Formation after Orthotopic Neobladder Cystectomy

**DOI:** 10.1155/2014/872989

**Published:** 2014-11-10

**Authors:** Zeng Shu-xiong, Zhang Zhen-sheng, Yu Xiao-wen, Li Hui-zhen, Lu Xin, Sun Ying-hao, Xu Chuan-liang

**Affiliations:** ^1^Department of Urology, Changhai Hospital, Second Military Medical University, Changhai Road No. 168, Yangpu District, Shanghai 200433, China; ^2^Department of Geriatrics, Changhai Hospital, Second Military Medical University, Shanghai 200433, China

## Abstract

*Introduction*. Laparoscopic and robot-assisted laparoscopic surgery are widely performed in urology field, so Hem-o-Lok clips are thus extensively used in the laparoscopic procedures. We describe the first case of Hem-o-Lok clip which migrated into the neobladder with calculus formation 26 months after laparoscopic orthotopic neobladder cystectomy, which causes symptoms of gross hematuria and frequent urination. *Case Presentation*. A 57-year-old man with T2a muscle invasive bladder cancer underwent laparoscopic orthotopic sigmoid neobladder reconstruction after cystectomy which was complicated by intestinal anastomosis leak and peritoneal abscess requiring transverse colostomy and drainage 15 days postoperatively. Twenty-six months after cystectomy, he complained of gross hematuria and frequent urination. Computerized tomography and plain pelvic X-ray revealed a stone measuring approximately 2.8 cm in diameter in the neobladder. During cystoscopy, a closed whitish Hem-o-Lok clip was seen in the center of the calculi. No anastomotic leak or neoplasm was found during cystoscopy. *Conclusion*. Hem-o-Lok clip migration into the bladder after laparoscopic orthotopic neobladder cystectomy is a rare complication; the first reported case in the literature. To prevent Hem-o-Lok clip migration, it is recommended to avoid extensive use of Hem-o-Lok clip close to anastomosis site, and any loose Hem-o-Lok clip should be retrieved before closure.

## 1. Introduction

Laparoscopic cystectomy with different kinds of urinary diversion is increasing being applied for patients with muscle invasive bladder cancer. Hem-o-Lok clip (HOLC) is thus extensively used in the procedures for hemostasis and tissue ligation in laparoscopic cystectomy. We present the first case of neobladder migration of HOLC with stone formation after laparoscopic orthotopic neobladder cystectomy.

## 2. Case Presentation 

**Figure 1:**
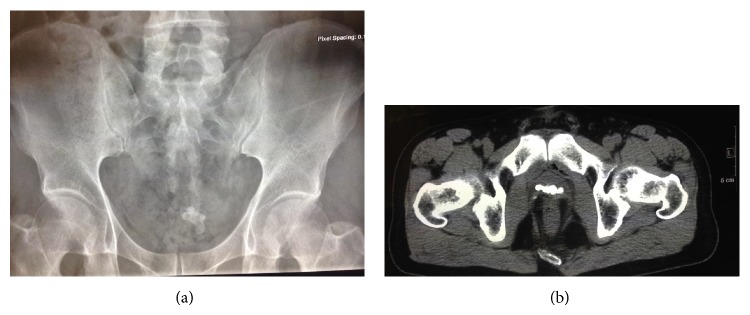
Pelvic X-ray (a) and computed tomography (b) demonstrate an approximately 2.8 cm calculus in the neobladder.

A 57-year-old man with T2a muscle invasive bladder cancer underwent laparoscopic orthotopic sigmoid neobladder reconstruction after cystectomy. During the operation, HOLC (Weck Surgical Instruments, Research Triangle Park, NC, USA) was used to ligate the lateral pedicle, and HOLC as well as titanium metal clip was utilized to control vein bleeding near proximal urethral stump if needed. Urethroneobladder anastomosis was performed with V-Loc self-retaining suture and intestinal anastomosis was done with PROXIMATE Linear Cutters Surgical Stapling. The operation was complicated by intestinal anastomosis leak and peritoneal abscess requiring transverse colostomy and drainage 15 days postoperatively. The patient then recovered uneventfully and had a satisfactory continence. Transversostomy was performed 6 months later. Twenty-six months after cystectomy, he complained of gross hematuria and frequent urination. Physical examination was unremarkable. Urinalysis showed leukocyturia and hematuria, but urine culture was negative. Computerized tomography and plain pelvic X-ray revealed a stone measuring approximately 2.8 cm in diameter in the neobladder (Figure 1).

**Figure 2:**
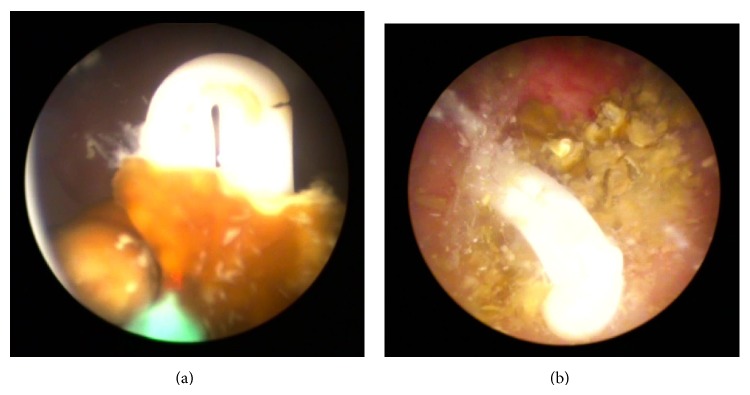
A Hem-o-Lok was encrusted by calculus (a); the intact Hem-o-Lok after the stone was fragmented (b).

Therefore, the patient was admitted for holmium laser cystolithotripsy. A F22 rigid cystoscopy was successfully inserted into the neobladder. During the procedure, an approximately 2 cm whitish HOLC clip was surprisingly found in the center of the calculi (Figure 2(a)). A closed and intact HOLC was seen after the stone was fragmented (Figure 2(b)). The neobladder and urethra were then carefully inspected after the HOLC and stone flushed out; however, no anastomotic leak or neoplasm was found. An indwelling urethral catheter was left for 1 day, and the patient was discharged the following day. The symptoms he complained of disappeared 3 days later.

## 3. Discussion

Laparoscopic or robot-assisted laparoscopic radical cystectomy is now widely performed for patients with muscle invasive bladder cancer. Thus, there has been an increasing interest in hemostatic alternatives to suture ligation, among which HOLC is the most commonly used device [1]. The HOLC was first introduced for vascular control in 1999, since then it has been extensively used in the laparoscopic surgery for its safety and reliability for vascular control [2, 3]. In the laparoscopic cystectomy, HOLC is used for ligation of vesical arteries, vas deferens, bilateral ureters, seminal vesicle arteries, and prostatic pedicles.

It was reported that the use of HOLC had been associated with some complications, the majority of which are related to hemorrhage [4]. Another concern is that HOLC may migrate into adjacent structures such as the bladder, ureter, and rectum, causing complications, for example, calculus formation, bladder neck contracture, urethral stricture, anastomotic leak, and lower urinary tract symptoms [4–8]. Migration of the HOLC is a rare complication; the overall incidence of HOLC migration is unknown. Blumenthal et al. [4] reported 3 cases of HOLC migration among 524 consecutive laparoscopic prostatectomies (3/524, 5.7‰). Yi et al. [9] observed 2 patients had suffered from bladder neck contracture caused by HOLC migration in 153 consecutive cases who had undergone robotic-assisted laparoscopic prostatectomy (2/153, 1.3%). After thoroughly searching the PubMed, we found 16 patients had suffered from HOLC migration among 10 published articles [1–10]. All of these patients had undergone laparoscopic or robotic-assisted laparoscopic prostatectomy. It was suggested to limit the use of HOLC near the vesicourethral anastomosis, and any loose HOLC should be retrieved [1, 3]. Furthermore, bipolar electrocautery should be applied carefully near the bladder neck and prostatic pedicles for minimizing inflammation [6].

To our knowledge, the case presented is the first reported case involving HOLC migrating into the neobladder with calculus formation after laparoscopic orthotopic neobladder cystectomy. The exact mechanism by which the HOLC in the present case made its way into the neobladder was elusive. However, HOLC most likely migrated into neobladder lumen through urethroneobladder anastomosis, several theoretical possibilities may attribute to this situation. Firstly, the presented patient had suffered from intestinal anastomosis leak and peritoneal abscess after cystectomy; inflammation may arise around the neobladder anastomosis, which may facilitate the HOLC eroding the anastomosis and eventually migration into the neobladder. Secondly, HOLC used in the pelvic may overlapped against the neobladder anastomosis, which may increase the pressure of HOLC on the anastomosis and eventually migration into the neobladder lumen. While this particular case represents a rare event after orthotopic neobladder cystectomy, surgeons should take HOLC migration into consideration when patients present intractable urinary tract symptoms; further computerized tomography or cystoscopy may be needed since HOLC was not picked up on by X-ray [8].

## 4. Conclusions

Hem-o-Lok clip migration into the bladder after laparoscopic orthotopic neobladder cystectomy is a rare complication; this case was the first reported case in the literature. To prevent HOLC migration, it is recommended to avoid extensive use of HOLC close to anastomosis site, and any loose HOLC should be retrieved before closure.
